# Lambda3: homology search for protein, nucleotide, and bisulfite-converted sequences

**DOI:** 10.1093/bioinformatics/btae097

**Published:** 2024-03-14

**Authors:** Hannes Hauswedell, Sara Hetzel, Simon G Gottlieb, Helene Kretzmer, Alexander Meissner, Knut Reinert

**Affiliations:** deCODE genetics/Amgen Inc., Reykjavik, Iceland; Department of Genome Regulation, Max Planck Institute for Molecular Genetics, Berlin 14195, Germany; Department of Mathematics and Computer Science, Freie Universität Berlin, Berlin 14195, Germany; Institute for Bio- and Geosciences, Forschungszentrum Jülich GmbH, Jülich 52428, Germany; Department of Genome Regulation, Max Planck Institute for Molecular Genetics, Berlin 14195, Germany; Department of Genome Regulation, Max Planck Institute for Molecular Genetics, Berlin 14195, Germany; Department of Biology, Chemistry and Pharmacy, Freie Universität Berlin, Berlin 14195, Germany; Department of Mathematics and Computer Science, Freie Universität Berlin, Berlin 14195, Germany; Efficient Algorithms for Omics Data Group, Max Planck Institute for Molecular Genetics, Berlin 14195, Germany

## Abstract

**Motivation:**

Local alignments of query sequences in large databases represent a core part of metagenomic studies and facilitate homology search. Following the development of NCBI Blast, many applications aimed to provide faster and equally sensitive local alignment frameworks. Most applications focus on protein alignments, while only few also facilitate DNA-based searches. None of the established programs allow searching DNA sequences from bisulfite sequencing experiments commonly used for DNA methylation profiling, for which specific alignment strategies need to be implemented.

**Results:**

Here, we introduce Lambda3, a new version of the local alignment application Lambda. Lambda3 is the first solution that enables the search of protein, nucleotide as well as bisulfite-converted nucleotide query sequences. Its protein mode achieves comparable performance to that of the highly optimized protein alignment application Diamond, while the nucleotide mode consistently outperforms established local nucleotide aligners. Combined, Lambda3 presents a universal local alignment framework that enables fast and sensitive homology searches for a wide range of use-cases.

**Availability and implementation:**

Lambda3 is free and open-source software publicly available at https://github.com/seqan/lambda/.

## 1 Introduction 

The approximate search of query sequences in large annotated databases is a central part of sequence analysis. Queries such as sequencing reads are aligned to a reference genome or a collection of subject sequences in order to detect regions that share different levels of sequence similarity with the query. Identifying exact or near-exact matches of the query sequence in the reference database is required during the process of read mapping, where the genomic origin of a sequencing read is detected. For this purpose, semi-global alignments are used that allow a limited amount of differing bases between the read and reference genome to account for potential technical errors or genetic variation within a species (Reinert *et al.* 2015). Additionally, in most cases only the best-scoring match is reported as sequencing reads originate from a single genomic location that needs to be correctly identified—except for repetitive or not assembled regions.

In other fields, such as homology search, it is beneficial to also identify more distant matches. Here, sequences of common evolutionary descent within or across species are determined, which plays a role in the identification of known and unknown species in contaminated or mixed background samples (Pearson 2013). Additionally, the relatedness of different species can be determined using taxonomic classifications based on homology search (Pearson 2013). This is particularly relevant for metagenomic or -transcriptomic studies, where samples are usually not associated with a single species but instead comprise a diversity of organisms (Tringe and Rubin 2005). In order to identify homologues across large reference databases, local alignments are used that can detect more distant matches of query (sub-)sequences that might be evolutionary conserved (Smith and Waterman 1981). In this context, it is also frequently desirable to identify not only one but many matches per query across the database in order to assess its distribution across species and thus facilitate taxonomic analyses (Pearson 2013).

Identifying a large number of local hits across many query and subject sequences represents a computational challenge that is typically addressed using heuristic algorithms. These are not exact, but ensure feasibility of such searches given the ever-growing size and amount of sequencing datasets. Additionally, and in contrast to read mapping, homology search can make use of protein instead of nucleotide alignments, which may reveal a different type of conservation that is not apparent from the DNA level (Pearson 2013). Protein alignments can also reduce the search space as the amount of known protein sequences is drastically smaller than the genomic counterparts, thus accelerating the search process. Previously, many local alignment applications have been developed of which Blast is the gold standard for protein and nucleotide searches. It also provided the statistical basis for many applications developed in the following years (Altschul *et al.* 1990), most of which greatly improved the speed compared to Blast (sometimes by a factor of over 1000). These include Lambda (Hauswedell *et al.* 2014) and malt (Vågene *et al.* 2018) for protein and nucleotide alignments, as well as Diamond that implements extremely fast and sensitive protein alignments (Buchfink *et al.* 2015, 2021).

Here, we introduce Lambda3, which improves the performance of previous Lambda versions, making it competitive with diamond’s fast protein alignments while additionally providing faster nucleotide searches than state-of-the-art applications. As a new feature that has not been provided by other local alignment applications so far, Lambda3 offers a mode that enables the search of queries from bisulfite sequencing experiments. Together, these advances make Lambda3 a fast and universal application for protein and nucleotide homology search.

## 2 Materials and methods

### 2.1 Application overview

**Figure 1.**
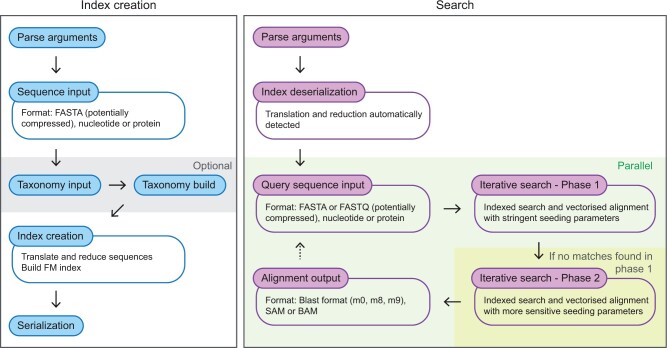
Overview of Lambda3. The program consists of two main parts, the index creation and the search of query sequences in the index.

Lambda3 operates in three distinct ‘domains’: nucleotide, protein, and bisulfite. Furthermore, the application is divided into two parts: the index creation and the search (Fig. 1). Sub-commands (similar to, e.g. the git program) exist for each part and domain, i.e. lambda3 mkindexn <arguments> and lambda3 searchn <arguments> for the nucleotide domain. Table 1 provides an overview of the sub-commands.

**Table 1.** The domain specific sub-commands available in Lambda3 and corresponding Blast programs.

Domain	Index creation	Search	Equivalent Blast programs
Protein	mkindexp	searchp	BlastP, BlastX, TBlastN, and TBlastX
Nucleotide	mkindexn	searchn	BlastN, MegaBlast
Bisulfite	mkindexbs	searchbs	—

The index creation involves reading the subject sequences (usually FastA) and computing an FM-index, a step that is computationally expensive but only has to be performed once. Optionally, Lambda3 can also parse and include taxonomic annotations, which allow subsequent searches to calculate the lowest-common ancestor (LCA) of all database matches detected for a single query. The index as well as associated options and annotations are stored in a single file on disk.

The search commands load the previously computed index, as well as the query sequences (reads). In contrast to previous versions of Lambda, the query is not indexed. Seeding is performed in the FM-index with various heuristic optimizations and parameters specific to each domain. For queries that have no promising hits after seeding, a second, more sensitive seeding step is performed. After seeding, hits are extended locally, scored and compared against the desired threshold (bit-score or e-value). Verified hits are written to an output file (Blast formats and Sequence Alignment Map (SAM) resp. Binary Alignment Map (BAM) supported).

It is important to note, that the program architecture for all domains is very similar, but that the choice of parameters and some algorithmic components differ (more on this below). In some cases, the domain implies specific sequence data as input, e.g. the nucleotide commands expect nucleotide sequences (similar to BlastN and MegaBlast). The protein commands, on the other hand, can be used with both protein and nucleotide data (for query and/or subject sequences). In this case, nucleotide sequences are automatically detected and translated to amino acids. This unifies all of Blast’s protein modes into a single, easy-to-use interface (see Table 1 for an overview).

### 2.2 Algorithm

#### 2.2.1 Input/output and user interface

Lambda3 is provided as a single command-line application with multiple sub-programs. Argument handling is provided by the Sharg-Parser library (Mehringer and Seiler 2023 at https://github.com/seqan/sharg-parser), and Lambda3 has a fully documented interface, including manual pages.

Sequence input files are read using the BioC++ I/O library (Hauswedell 2023 at https://github.com/biocpp). Both FastA and FastQ formats are supported, including compressed versions (bgzip, gzip, bzip2). The query file can either be loaded completely at program start (default), or is lazily read by a background thread. The latter option can save memory if the query file is larger than the index, but it uses one of the CPU threads exclusively for I/O.

Taxonomic annotations (NCBI or UniProt format) are handled using a custom parser and can be used to annotate subject sequences. The output of the indexer sub-command includes the FM-index, original subject sequences and identifiers, taxonomic annotations (if provided) and corresponding parameters. All of these are written to a single (binary) archive file and later read by the search command, using the cereal library (Grant and Voorhies 2023 at https://github.com/USCiLab/cereal). This is an improvement compared to previous versions of Lambda, where multiple files were generated and stored in a separate output directory. Optionally, the index file can be compressed, which reduces the file’s size by 36% (tested using the UniRef50 database).

The final search results can be written to disk in Blast formats (tabular or pairwise) or SAM/BAM using the SeqAn2 library (Reinert *et al.* 2017).

#### 2.2.2 Data storage and alphabets

The two main data sources in the application are the query and the subject sequences. Three different representations (transformations) of these sequences are used in different parts of Lambda3.

##### 2.2.2.1 Original sequences

These are the sequences as provided by the user. Depending on the domain, they can be nucleotides or amino acids. Amino acids are stored using the 27-letter alphabet provided by the BioC++ core library (aa27), which includes the 20 canonical amino acids, as well as rare and ambiguous letters. For nucleotides, the 5-letter DNA alphabet (dna5: A, C, G, T, N) is used. RNA alphabets are supported as input, but all U letters will be treated as T. In the protein domain, the query and subject sequences might have different alphabets (such as nucleotide query sequences and protein subject sequences in BlastX mode).

##### 2.2.2.2 Translated sequences

As the name implies, these always refer to sequences in the protein domain. If the original sequences are nucleotides, Lambda3 translates these into all six possible reading frames. In case of protein input sequences, the translated sequences are identical to the original sequences. In the nucleotide and bisulfite domains, the translated sequences always have the same alphabet as the original sequences, but a reverse complement ‘frame’ is added for every query sequence. In the bisulfite domain, both query and subject sequences are additionally duplicated to allow for separate handling of queries originating from different strands (see below).

##### 2.2.2.3 Reduced sequences

These are built from the translated sequences and transform the alphabet to a new (typically smaller) alphabet. In the protein domain, a functional reduction based on biochemical amino acid properties is implemented (see section 2.2.3 for details). In the nucleotide domain, sequences are reduced to a 4-letter alphabet (dna4) by replacing unknown bases (N) with a random base (A, C, G, or T). This allows creating a smaller and faster FM-index. See section 2.2.4 for the transformation applied in the bisulfite domain.

An overview of the alphabets and frames is shown in Table 2. The different algorithmic components in Lambda3 work on different types of sequences. In particular, the search and seeding steps operate on the reduced sequences to increase performance and sensitivity. In contrast, the alignment step operates on the translated, unreduced sequences in order to ensure specificity. Using BioC++ and C++ Ranges, only the original sequences are ever stored in memory, and both the translated and reduced sequence sets are created as so-called views (Hauswedell 2022). Views behave like containers, allowing constant-time, non-allocating random access. However, the elements (both individual characters and entire sequences) are generated lazily when accessed. This has no measurable performance overhead compared to generating and storing the additional datasets, but saves memory, and allows for a clean program architecture.

**Table 2.** The alphabets used and frames generated in the respective domains of the program.^a^

	Original	Translated			Reduced
Domain	Alphabet	Alphabet	QF	SF	Alphabet
Protein	aa27/dna5	aa27	1/6	1/6	aa27, li10 or mu10
Nucleotide	dna5	dna5	2	1	dna4
Bisulfite	dna5	dna5	4	2	6-letter BS alphabet

a QF/SF are the number of frames generated per query/subject sequence. Input alphabets in protein domain are detected from input; nucleotide leads to six-frame translation. Reduced alphabets in protein domain depend on user choice (li10 is default).

#### 2.2.3 Protein reduction

Reduced amino acid alphabets are a common feature of protein aligners (Liang *et al.* 2022). A reduced amino acid alphabet is a small(er) alphabet, where groups of amino acids are each represented by a single letter. They are created by clustering amino acids by various biochemical properties or substitution probabilities.

The main purposes of employing a reduction are:

1. Enable matching of (functionally) similar amino acids to account for likely mutations. This improves the sensitivity of matches.
2. Improve the performance of algorithmic steps. A smaller alphabet results in smaller and more efficient data structures, in particular the FM-index.

**Figure 2.**
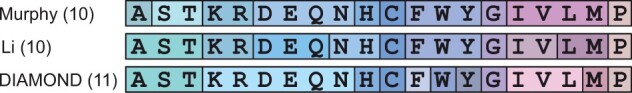
Overview of different amino acid alphabet reductions. For simplicity, non-canonical amino acids and special characters are omitted here. MALT uses the same alphabet as DIAMOND.

The exact criteria for clustering different amino acids into one letter, as well as the target size of the reduced alphabet, vary. Many different reduced alphabets exist in the literature. For protein aligners, alphabets of size 10–12 have been popular, and previous Lambda versions used the reduction by Murphy *et al.* 2000 (mu10). In Lambda3, the new default reduced amino acid alphabet is the one proposed by Li *et al.* (2003) (li10). An overview of the amino acid clusters defined by the different alphabets are shown in Fig. 2.

Depending on the query dataset and database, reduced alphabets can perform differently in regard to sensitivity (number of valid results) and performance. We evaluated Diamond’s alphabet and found it to be sensitive but not very fast in our application; it is likely that these properties are balanced out by other parameters and heuristics in Diamond’s algorithm. For Lambda3, Li’s alphabet shows an improved performance while maintaining a similar sensitivity to Murphy’s. Users who favor sensitivity over speed—especially with very small datasets—have the option to continue using Murphy’s alphabet via a command-line argument.

#### 2.2.4 Bisulfite reduction

**Figure 3.**
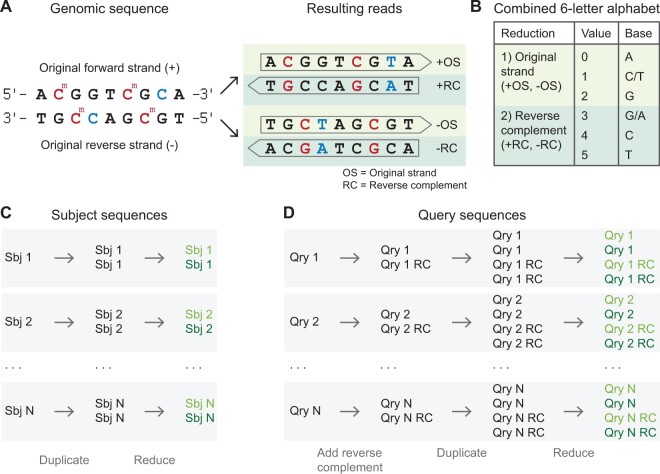
Search of bisulfite-converted sequences using a 3-letter alphabet. (A) Sodium bisulfite treatment leads to the conversion of unmethylated cytosines to uracils. During subsequent amplification steps, uracils get replaced with thymines. As a result, four different read types can be distinguished that stem from the original forward and reverse strand or their reverse complements. (B) In order to build a single index for bisulfite-aware alignments, Lambda3 implements an artificial 6-letter alphabet, where the first three letters represent the reduced alphabet associated with reads from the original strands (C and T are considered identical). The last three letters represent the reduced alphabet for reads originating from the reverse complements of the original strands (G and A are considered identical). (C) Before the index is built, subject sequences are duplicated. The first copy is subsequently reduced using the first three letters of the 6-letter alphabet, while the second copy is reduced using the last three letters. This way, the index is built across the same alphabet that accounts for both reductions without the necessity to build two indexes. (D) Query sequences are reduced analogously to the subject sequences after the sequences have been reverse complemented to account for every possible alignment according to the four read types.

Sodium bisulfite treatment of the DNA leads to the conversion of unmethylated cytosines to uracils, which are replaced with thymines in subsequent amplification steps (Frommer *et al.* 1992). Sequencing of the resulting fragments allows determining the DNA methylation status of cytosines in the genome. Depending on the protocol and the sequencing setup, this can lead to four different read types, which arise from the bisulfite conversion itself (reads reflecting the original forward and reverse strand) or the subsequent amplification, which includes the reverse complements of the original strands (Fig. 3A, Cokus *et al.* 2008). Due to the bisulfite conversion, the reverse complement of the original forward strand does not equal the original reverse strand as for standard DNA sequencing.

To account for the effects of bisulfite conversion, the search and alignment process need to be adapted: C (subject) to T (query) mismatches need to be considered as matches for reads originating from the original forward or reverse strand of a DNA fragment. Similarly, G (subject) to A (query) mismatches should not be penalized for reads originating from the reverse complements of the original strands (Fig. 3A). In order to account for this, we make use of a common concept that has been established for many bisulfite alignment applications (Kunde-Ramamoorthy *et al.* 2014). Here, the actual search is carried out using one of two 3-letter alphabets, where either C and T or G and A are considered as identical.

For Lambda3, we implemented an artificial 6-letter alphabet where the first and the last three letters represent the two different reduced 3-letter alphabets respectively (Fig. 3B). The subject sequences are duplicated prior to the index construction. One copy is reduced according to the first three letters of the new alphabet, while the second copy is reduced according to the last three letters. This offers the possibility to search reads that originate both from the original strands and their reverse complements (Fig. 3C). For the query sequence, first the reverse complement is added (similar to the nucleotide domain, translated sequences) and then treated analogously to the subject sequences (reduced sequences, Table 2 and Fig. 3D).

Using a combined alphabet for both bisulfite reductions has the advantage that a single index can be built. Therefore, the general application architecture remains the same as in the other domains. Other alignment applications typically generate two indexes with different alphabet reductions, and queries are subsequently searched in both data structures (Krueger and Andrews 2011, Otto *et al.* 2012).

#### 2.2.5 Seeding

The initial search is performed using an FM-index (Ferragina and Manzini 2000), which is implemented in the standalone FMIndex-Collection library (Gottlieb 2023, see https://github.com/SGSSGene/fmindex-collection). The advantages of FM-indexes over hash-tables are inexact searches and a runtime choice of the seed length, while hash-tables allow asymptotically faster look-ups. Bidirectional FM-indexes are supported by Lambda3, but are not used by default as they did not outperform the unidirectional index combined with its heuristics (see below).

The index is always built on the reduced subject sequences to enhance performance and sensitivity. Multiple worker threads run in parallel, and each thread takes chunks of query sequences. The sequences in each chunk are translated and reduced (depending on the domain and user settings), split into seeds and searched in the index. Promising hits are then handed over to the alignment step.

**Figure 4.**
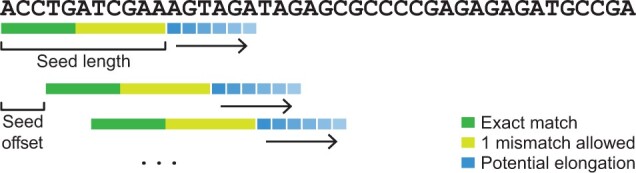
Lambda3’s seeding strategy. Seeds are defined by their length and the offset between start positions. By default, an error is only considered in the second half of the seed. If too many hits are found, the seed is automatically extended.

Seeding is determined by many parameters, that each influence sensitivity versus speed (Fig. 4). The most important ones are the minimum ‘seed length’, the distance between seed begin positions (‘seed offset’, determines overlap of seeds), and whether a mismatch is allowed in the seed (‘seed delta’, 0 or 1). These parameters can be chosen by the user, and their default values depend on the domain. Additionally, predefined profiles with faster or more sensitive parameters are available.

In combination with these common parameters, several heuristics are employed. ‘Iterative search’ is defined as trying a fast search first, and performing a more sensitive search only if the first yielded no promising results. The benefits of this option depend strongly on the dataset and the expected success-rate of the search. ‘Adaptive seeding’ represents the dynamic elongation of the seed size if the seed is found too often. This is based on the assumption that seeds that map to too many locations are less relevant due to their universal occurrence. Another advantage of the seed expansion is the acceleration of the subsequent alignment step, because fewer hits need to be considered. ‘Half-exact seeding’ allows the seed delta only in the second half of the seed in order to reduce search complexity. The loss in sensitivity can usually be compensated by a smaller seed offset (higher overlap).

Alphabet reduction can also be classified as a form of heuristic, and, depending on the domain, may introduce many false positive hits. To remove these from downstream analyses, every potentially matching region is evaluated in the unreduced sequence space via a fast, gapless local alignment.

#### 2.2.6 Alignment and scoring

After a worker thread has searched seeds for all its query sequences, the resulting matches are sorted and distributed into batches of similar sequence length. These are then extended via a gapped local alignment of the entire unreduced query sequence against the target region of the subject sequence. Each thread computes multiple alignments at the same time through Single Instruction Multiple Data (SIMD) acceleration (Rahn *et al.* 2018). In contrast to previous versions of Lambda, no X-drop heuristic is used, as this would prevent vectorization. This implies that the current algorithm is optimized more strongly for short reads. In the bisulfite mode, matches are sorted additionally based on the reduction of the subject sequence that the query mapped to (first or second half of the combined 6-letter alphabet, Fig. 3B and C). The alignment process is then carried out separately for the two reduction versions in order to account for the different types of conversion effects. For all matches that were found in subject sequences that treated C and T bases as equal, an imbalanced scoring-scheme is used that allows C (subject) to T (query) mismatches but penalizes T to C mismatches. Similarly, for matches found in subject sequences that considered G and A as the same base, only G (subject) to A (query) mismatches are not penalized. This reduces the false positive rate arising from the search in a 3-letter alphabet.

At the end of the alignment step, every alignment is scored, and an output record is generated if it passes the respective thresholds. Lambda supports e-value cut-offs, bit-score thresholds and/or a minimum percent identity. The bit-score is derived from the alignment score by incorporating constants derived from observed substitutions in empirical studies and parameters of the used scoring-scheme (Altschul *et al.* 1990, Karlin and Altschul 1990). This allows comparing bit-scores between different scoring-schemes and domains. Higher bit-scores indicate better alignments. E-values are derived from the bit-score by taking into account the length of the query sequence and the total size of the database. They are a measure of significance, where an e-value of 1 indicates that, given the query sequence length and scoring-scheme, one alignment is assumed to be found by chance in the database. Smaller e-values indicate more significant alignments; the same alignment found in databases of different sizes will have different e-values (but the same bit-score).

## 3 Results

### 3.1 Benchmark setup

Informative and reliable benchmarking of local alignment applications is not trivial, because use-cases vary strongly, and various applications with many tunable parameters exist. To this date, no other application covers all the domains that are covered by Lambda3, but some cover both the protein and nucleotide domain. Instead of the previously published version of Lambda (Hauswedell *et al.* 2014), we compared against Lambda2 (version 2.0.1) which is the most commonly used Lambda branch prior to the release of Lambda3. The ‘gold standard’ application Blast was omitted from our comparisons, simply because it cannot be run feasibly on the data sizes used. However, comparisons on much smaller datasets (and including Blast) are provided in the Supplementary information. Furthermore, existing research has already established that the discussed applications operate in a similar general sensitivity range—even if they are slightly less sensitive in their default settings (Hauswedell *et al.* 2014, Buchfink *et al.* 2015).

We are aware that applications perform differently on different inputs, and that the choice of files influences the perceived performance strongly. Therefore, we selected a variety of query datasets, both simulated and real-world, and including DNA and RNA sequencing experiments. Where available, we chose well-established databases, or created reference datasets in line with previous studies and potential use-cases.

There are different ways to measure sensitivity. Lambda and other applications produce more than one hit per query (the desired upper bound can be set via the command line), and there are several use-cases for utilizing these secondary alignments, especially in taxonomic analyses. However, there are also use-cases where these are not relevant, and it is not trivial to compare secondary alignments between applications. Simply counting them is not meaningful, as this hides their distribution between different query sequences. Ambiguities also arise from matches that appear as one long alignment in the output of one application and as two separate smaller ones in the output of another; the single longer result would usually be preferable, so a higher total count might even indicate unfavorable results. Therefore, we chose the widely used metric of the number of query sequences with at least one hit (Huson and Xie 2014, Ye *et al.* 2011). This is a lower bound for the sensitivity, because not finding any results for a query sequence is clearly detrimental to all further analyses. Additionally, we provide a separate benchmark specifically for the task of maximizing the number of query-subject pairs detected (supplementary information). All results that pass the e-value and/or bit-score threshold are considered true positives (except in the bisulfite domain, see Supplementary information).

We chose an e-value cut-off of 0.01 for our analyses, which implies that less than 1% of the reported results are expected to have occurred by chance. The cut-off is lower than Blast’s default, but slightly higher than what we used in previous analyses (Hauswedell *et al.* 2014), as we found that applying a more stringent cut-off removes many high-scoring alignments that were previously reported with smaller databases. As elaborated previously, the method of e-value calculation between different applications is not reliable, resulting in different e-values for the same alignment (Hauswedell *et al.* 2014). Bit-scores, on the other hand, seem to be the same for identical alignments reported by multiple applications. To improve comparability, we pre-computed the bit-score equivalent of the 0.01 e-value cut-off for every query and subject dataset combination and used the resulting bit-score threshold instead. We used the simplest and most widely accepted formula of $\mathrm{bitScore}=log_{2}(\frac{m*n}{\mathrm{eValue}})$, where *m* represents the query length and *n* the total size of the database. This ignores certain statistical fine-tuning that some applications may or may not employ, but as long as the same bit-score threshold is used for all applications, the method of deriving such a threshold should favor no application over another.

All benchmarks were performed on a dual-socket system with two Intel(R) Xeon(R) Gold 6248 CPUs (each provide 20 cores and can execute 40 threads), one terabyte of RAM and regular hard drives. The query datasets are sampled to be exactly 200 MiB big, and the applications are configured to use up to 40 threads.

### 3.2 Protein domain

#### 3.2.1 Datasets

Searching translated nucleotide data in a protein database is the most-common form of protein search, also known as BlastX. We selected two simulated datasets (q1 and *q2* in Table 3) by the Initiative for the Critical Assessment of Metagenome Interpretation (CAMI) which offers comprehensive datasets to enable benchmarking of applications applied in metagenomic studies (Meyer *et al.* 2022). Additionally, we selected two real-world sequencing datasets: a topsoil DNA sample (*q3*) and an RNA sequencing experiment of a human colorectal tumor (including the associated gut microbiome; *q4*). Both datasets were generated as part of metagenomic studies (Bahram *et al.* 2018, Visnovska *et al.* 2019). We used UniRef50 (downloaded 25 May 2022) as the database for all protein searches, which is also the reference database used by Buchfink *et al.* (2021).

**Table 3.** Query datasets used to evaluate the performance of Lambda3 and comparable tools.

ID	Query set	Length (bp)	Molecule	Source
q1^a^	Strain diversity	150	DNA (simulated)	CAMI II challenge (2022)
q2^a^	Plant-associated	150	DNA (simulated)	CAMI II challenge (2022)
q3	Topsoil	251	DNA	Bahram *et al.* (2018)
q4	Colorectal tumor (gut microbiome)	125	RNA	Visnovska *et al.* (2019)
q5^a^	Strain diversity	150	DNA (simulated, *in silico* bisulfite-converted)	CAMI II challenge (2022)
q6^a^	Plant-associated	150	DNA (simulated, *in silico* bisulfite-converted)	CAMI II challenge (2022)
q7	Xenograft breast tumor	125	Bisulfite-converted cell-free DNA	Liu *et al.* (2021)
q8	Fungi	76	Bisulfite-converted DNA	Bewick *et al.* (2019)

a Datasets of the CAMI challenge were additionally *in silico* bisulfite-converted in order to test Lambda3’s bisulfite mode.

#### 3.2.2 Applications

We ran Lambda3 (commit d995cb5, after the 3.0.0 release) and Lambda2 (version 2.0.1) in its default mode and the predefined ‘fast’ and ‘sensitive’ profiles (for Lambda2, these profiles are provided as recommended settings). For Lambda2, the desired number of hits per query was reduced from 256 to 25 to be in line with the default values of the other applications. Diamond (version 2.1.6) was run with its default, fast and mid-sensitive profiles, as these corresponded most closely to the profiles benchmarked for Lambda3. More sensitive profiles are available; however, these operate in an entirely different region on the speed ↔ sensitivity spectrum (Buchfink *et al.* 2021). Lastly, we ran malt (version 0.6.1) in its default mode (no fast or sensitive profiles are available). All applications by default use the Blosum62 matrix for scoring, combined with a score of −1 for each gap-character and an additional cost of −11 for each contiguous sequence of gaps. To establish comparability of the scores, composition-based statistics were disabled if available (not all applications implement these adjustments and several incompatible implementations exist, Schäffer *et al.* 2001, Yu and Altschul 2005). The bit-score threshold was determined as 47 for q1, q2, and q4, as well as 48 for q3 (due to the longer query sequences).

#### 3.2.3 Results

**Figure 5.**
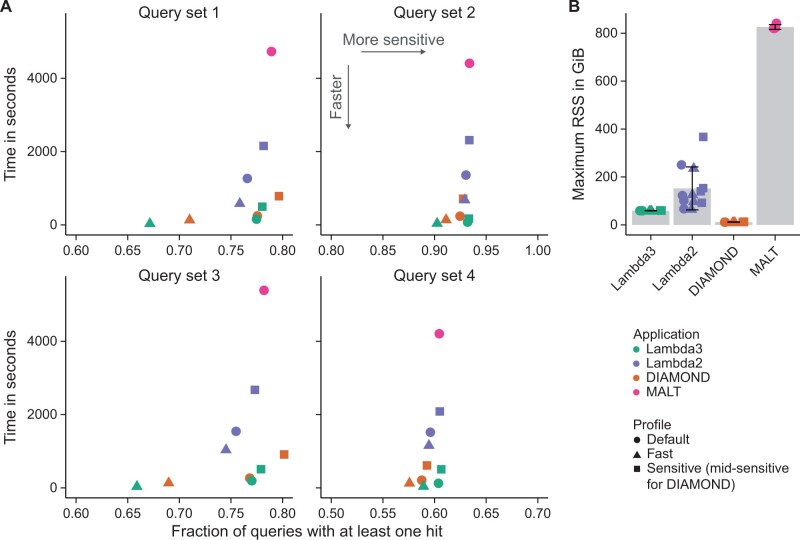
(A) Comparison of local alignment applications for protein search based on runtime and the fraction of detected query sequences of the fastest out of three runs are shown. (B) Memory consumption. Bars indicate the mean, and error bars indicate the standard deviation across all query datasets and profiles.

Every application’s default mode yields results in a similar overall sensitivity range (∼5%), but the speed varies significantly, and Lambda3 and Diamond consistently deliver the best results per time (Fig. 5A, shapes indicate modes that are expected to operate in the same sensitivity range). Lambda3’s profiles are on average twice as fast as the corresponding profiles of Diamond, while—depending on the query dataset and profile—Lambda3 or Diamond detect slightly more queries than the other. Although MALT exhibits similar sensitivity compared to Lambda3 and Diamond, it is on average 38 times slower than Lambda3 in its default mode, and it demands more than 800 GiB of RAM (Fig. 5A and B). In contrast, Lambda3 requires around 60 GiB—a notable improvement over Lambda2 that consumes between 67 and 250 GiB in the default mode. Diamond requires only 11 GiB (Fig. 5B). When speed is the primary concern, Lambda3’s fast mode is the clear choice, being the fastest in every comparison, often by a factor of three over Diamond’s fast and Lambda3’s default mode. However, it also misses up to 14% of the results in one dataset.

### 3.3 Nucleotide domain

#### 3.3.1 Datasets

For our benchmarks of the nucleotide domain, the same query datasets are used as in the protein domain. The database encompasses a collection of microbial genomes assembled by the Human Microbiome Project (downloaded 7 June 2022, Human Microbiome Project Consortium 2012).

#### 3.3.2 Applications

In line with the protein domain, Lambda3 and Lambda2 were compared with their default, fast and sensitive profiles using a maximum of 25 hits per query. Similarly, we ran MALT with its default mode. For the nucleotide domain, we also selected MegaBlast (version 2.13.0, Camacho *et al.* 2009), which represents a faster version of BlastN and was run in default mode. Since MegaBlast does not offer the option to filter results based on a bit-score threshold, we performed a first search with a relaxed e-value cut-off of 1 and subsequently determined the maximum e-value associated with the estimated bit-score thresholds. The actual benchmarks were then performed using these e-values. All applications were configured to use a scoring-scheme of {2,−3,−2,−5} (match, mismatch, gap, gap-open), which is already the default for most applications. The bit-score threshold was determined as 46 for q1, q2 and q4, and 47 for q3. These thresholds are reduced in comparison to the protein domain due to the smaller database size (six compared to 15 billion characters).

#### 3.3.3 Results

**Figure 6.**
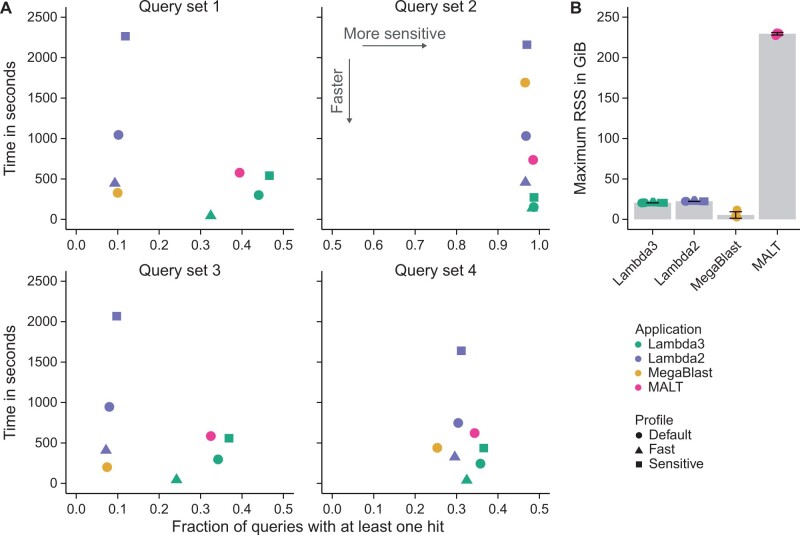
(A) Comparison of local alignment applications for nucleotide search based on runtime and the fraction of detected query sequences of the fastest out of three runs are shown. (B) Memory consumption. Bars indicate the mean, and error bars indicate the standard deviation across all query datasets and profiles.

Across all datasets, Lambda3 exhibits the highest sensitivity with its default and sensitive profiles (Fig. 6A). For most comparisons, Lambda3’s default mode also exhibits faster runtimes compared to the other applications, while its fast profile consistently outperforms all applications regarding the search speed. Lambda2 is always less sensitive and slower than Lambda3. For some datasets, it yields only a quarter of the results and is more than three times slower at the same time (Fig. 6A). MALT is of comparable sensitivity to Lambda3’s default profile, but between two and five times slower. Additionally, it requires more than 10 times as much memory (230 GiB versus 21 GiB, Fig. 6B). MegaBlast is in a similar speed range as Lambda3—in contrast to regular BlastN, which is orders of magnitude slower (Supplementary information). However, it misses 75% of the results that Lambda3 finds for query dataset q1. Its memory requirements, on the other hand, are the least demanding (less than 12 GiB, Fig. 6B).

### 3.4 Bisulfite domain

#### 3.4.1 Datasets

We *in silico* converted the CAMI datasets introduced above into sequences mimicking a bisulfite conversion experiment where cytosines were converted to thymines using a bisulfite conversion rate of 99% (scripts obtained from Nunn *et al.* 2021). For simplicity, we converted all query sequences accordingly, which reflects the effect of bisulfite conversion of reads from the original strands (Fig. 3A). To ensure that Lambda3 can also detect reads from reverse complements of the original strands, we additionally created a version of the two datasets where guanines were converted to alanines with the same conversion rate, which led to comparable results. These two query datasets are searched in the same database as in the nucleotide search. A common application for bisulfite sequencing is the read-out of cell-free DNA, which can reflect disease states such as tumors that can be identified using DNA methylation patterns, but has also been reported to contain fragments of microbial DNA (Legendre *et al.* 2015, Kowarsky *et al.* 2017). We therefore selected a breast tumor xenograft model bisulfite sequencing dataset, where the cell-free DNA of the xenograft model is expected to contain DNA fragments of both organisms (the host mouse model and the engrafted human cells), but also potential remnants of microbes (Liu *et al.* 2021). These reads are searched in a database consisting of the human (hg19) and mouse (mm10) genomes, as well as the Human Microbiome Project. As the fourth query file, we sampled a pan-fungi dataset from a study that profiled different fungi species in order to mimic a cross-species sequencing experiment (Bewick *et al.* 2019). All fully assembled fungi reference genomes (download 7 June 2022) were used as database.

#### 3.4.2 Applications

Lambda3 was run in its default mode and with its two predefined profiles (‘fast’ and ‘sensitive’). Lambda2 has no corresponding bisulfite mode and, therefore, was not considered for the benchmark. Since no local aligner for this type of queries has been developed to date, we chose the semi-global alignment applications gem3 (version 3.6.1), bsmap (version 2.90), and Bismark (version 0.24.0). Bismark does not offer the option to limit the number of threads reliably. The user can only specify the number of instances of Bismark that will be started in parallel that, according to the manual, start between two and six threads each. Therefore, the number of parallel instances was set to eight to approximate 40 threads. Bismark also offers a local mode, which we tested in addition to the semi-global alignment mode. GEM3 by default switches to a local mode if no alignments are encountered for a certain query. For Lambda3’s bisulfite domain, we needed to develop a new method to determine the bit-score threshold, because we expect different random distributions of hits due to the effects of the bisulfite conversion (compared to the nucleotide mode), and no respective predefined constants exist to normalize the raw alignment scores. The method is described in the Supplementary information. We attained the following thresholds: 68 (q5–q7) and 66 (q8). It should be noted that the other applications compared in our analysis perform no significance evaluation at all, so care should be taken when comparing the sensitivity. This implies that some of the other applications’ results are likely not significant, and that any kind of threshold will be unfavorable to Lambda3 in this comparison.

#### 3.4.3 Results

**Figure 7.**
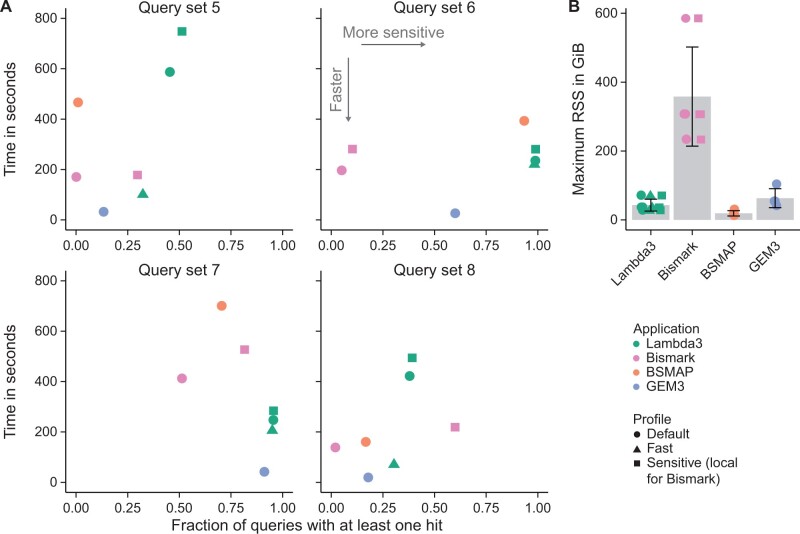
(A) Comparison of Lambda3’s bisulfite domain with semi-global bisulfite alignment applications based on runtime and the fraction of detected query sequences of the fastest out of three runs are shown. (B) Memory consumption of all runs shown in (A). Bars indicate the mean and error bars indicate the standard deviation across all query datasets and profiles.

Lambda3’s bisulfite mode consistently outperforms semi-global alignment applications based on the number of queries detected, except for q8, where Bismark’s local mode detects more queries (Fig. 7A). However, no program other than Lambda3 performs well consistently, e.g. Bismark detects almost no results for dataset q6. The default and sensitive profiles of Lambda3 are in some cases slightly slower than the semi-global aligners, which can be expected due to the larger number of seeds and hits that need to be processed. The fast mode shows comparable runtimes to BSMAP and Bismark while still detecting more queries for most datasets (Fig. 7A). GEM3 is the fastest application (its runtime depends mostly on the time required to load the database). Of all applications, Bismark consumes the most memory (up to 585 GiB for q3 with the largest database), while GEM3 and Lambda3 only use up to 103 GiB and 71 GiB of RAM, respectively (Fig. 7B). Lambda3’s bisulfite mode uses more memory than the corresponding nucleotide mode, which is expected due to the doubling of the number of subject sequences (Figs 6B and 7B). BSMAP is the most memory-efficient application (30 GiB for the largest database), which could be attributed to the fact that it is the only application that does not build an FM-index but instead is based on hash-tables (Fig. 7B).

## 4 Discussion

We presented Lambda3, a new version of the Lambda local alignment software that is more sensitive, faster, and requires less memory than previous versions. It is highly competitive with other modern applications in the protein and nucleotide domains, and it provides a novel mode to align bisulfite-treated sequences that is more sensitive and reliable than the semi-global applications previously available.

MALT, the only other local alignment application in our comparison that computes both nucleotide and protein alignments, is notably slower than Lambda3 in both domains and requires more than 10 times as much memory. Based on our observations, we see no advantage to using it in either domain. For many of the datasets tested, Lambda3 even outperforms the highly optimized protein aligner, Diamond—sometimes by a notable margin. However, we acknowledge that Diamond may be more suited when dealing with long read data or if the number of query sequences becomes much larger than what we tested (Diamond claims sublinear scalability while other applications, including Lambda3, scale linearly in runtime with the number of input sequences). In the nucleotide domain, there are fewer established local aligners, and Lambda3 seems preferable to all other compared applications, its default mode beating MegaBlast in speed and sensitivity on all tested datasets.

Our benchmarks showcased that the bisulfite domain of Lambda3 consistently detects more queries compared to standard semi-global alignment applications, which was most pronounced for actual metagenomic datasets (q5 and q6). These results show that established bisulfite alignment applications are not suitable for performing this type of search, even though some of them perform well in a query-dependent fashion. Therefore, Lambda3 represents the first application to reliably compute local alignments and could support future metagenomic studies using bisulfite sequencing.

We conclude that Lambda3 is a significant upgrade over previous versions. It stands out by being an integrated application that covers multiple data domains (protein-, nucleotide-, and bisulfite-treated data), and it exhibits very low runtimes, even with huge databases. Lambda3 provides many small but useful features, e.g. support for taxonomic binning and a variety of input and output formats (including SAM and BAM). Although short reads are still the dominant technology, we may focus on adding optimizations for long read data (e.g. support for frame-shift alignments) in the future. Integrating a technology like the DREAM index (Dadi *et al.* 2018, Seiler *et al.* 2021), would allow searching even larger databases.

## Supplementary Material

btae097_Supplementary_Data

## Data Availability

The source code underlying this work as well as data used for benchmarking are available at https://github.com/seqan/lambda/.
