# Supplementary material for: Lambda3: homology search for protein, nucleotide, and bisulfite-converted sequences
Source: Bioinformatics. 2024 Mar 14;40(3):btae097. doi: 10.1093/bioinformatics/btae097 (PMC10955267; doi:10.1093/bioinformatics/btae097)
Supplement: btae097_Supplementary_Data [file btae097_supplementary_data.pdf]

Supplementary information

S1 Configuration of the bisulfite mode

The effects of the bisulfite conversion have an impact on the local alignment scoring that requires adjusting the bit-score thresholds compared to the nucleotide domain. C to T (or G to A) mismatches arising from the bisulfite conversion are indistinguishable from actual mismatches of the same kind introduced, for example, via single nucleotide polymorphisms or larger differences in the genomic sequences of different species. Therefore, the bisulfite mode will generally lead to higher alignment scores and thus higher bit scores and lower e-values when using the same scoring parameters as the regular nucleotide mode. These scoring parameters are derived from experimental data and are used to normalise the raw alignment scores (Karlin and Altschul [1990], Altschul et al. [1990])—however, no such pre-calculated parameters exist for bisulfite searches, and we do not have access to sufficient data to derive them. Thus, the same parameters are used as in the nucleotide domain, but different thresholds need to be selected.

Even with a stricter significance threshold, we expect an increase in the overall false positive rate compared to regular nucleotide searches, because bisulfite conversion implicitly removes distinguishing information from the sequences. This likely has a higher impact for (shorter) local alignments than semi-global alignments.

Parameter selection We chose to select parameters including the bit-score threshold for the bisulfite mode in comparison to the regular nucleotide mode of Lambda3 and BlastN (version 2.13.0). For this purpose, we made use of the CAMI data sets (q1 and q2), of which we created *in silico* bisulfite-converted versions (q5 and q6). Since the bisulfite mode also performs a type of nucleotide search, we aimed to generate results similar to that of the regular nucleotide mode. The larger number of hits that arise due to the reduced alphabet complexity required the selection of less sensitive seeding parameters than for the nucleotide mode to enable sufficient performance and reduce the false positive rate. However, tightening the filtering criteria not only decreases the false positive hits but can also lead to missed true positives.

To evaluate whether the overall results detected by the bisulfite mode with different parameters are valid, we used the results of BlastN as ground truth. Even though its runtime made it unsuitable for our performance benchmarks, it still represents the gold standard application for local alignment sensitivity, especially in the nucleotide domain. This approach is an approximation as it is not guaranteed that a query that BlastN cannot detect represents an actual negative (i.e., a query that has truly no meaningful hits in the database). Therefore, we used BlastN with the same scoring scheme as Lambda3 and a relaxed cut-off of $e < 1$ to define an inclusive set of positive hits with the assumption that any query that cannot be detected with such a cut-off would be likely a false positive if detected by other tools. We then selected seeding parameters and the bit score threshold for Lambda3's bisulfite mode such that the true positive rate was comparable to Lambda3's nucleotide mode with respect to BlastN results while minimizing the false positive rate (Figure S1). Here, Lambda3's nucleotide mode was run with the bit-score threshold equivalent to the e-value cut-off 0.01 based on the query sequences length and database size for q1 and q2. True positives were defined as queries for which a hit was detected by both BlastN and Lambda3 while false positives were defined as queries for which only Lambda3 detected a hit, not BlastN. In addition, we measured the false discovery rate $FDR = \frac{FP}{FP+TP}$ to assess the fraction of false positives within the overall results of Lambda3's bisulfite mode.

Comparison with other applications We also calculated the true and false positive rates for the semi-global bisulfite alignment applications. Lambda3's bisulfite and nucleotide modes exhibit comparable and the highest true positive rates (default and sensitive profile). Additionally, the local nucleotide alignments performed by Lambda3 and MALT exhibit very low false positive and discovery rates. Similarly, the semi-global alignment applications present very low false positive rates, but true positive rates are often comparatively decreased (sometimes close to zero). In comparison, Lambda3's bisulfite mode shows true positive rates comparable to and sometimes exceeding Lambda3's and MALT's nucleotide mode (Figure S1). As expected, the false positive rates increase for the bisulfite mode but are comparable to the local alignment mode of Bismark.

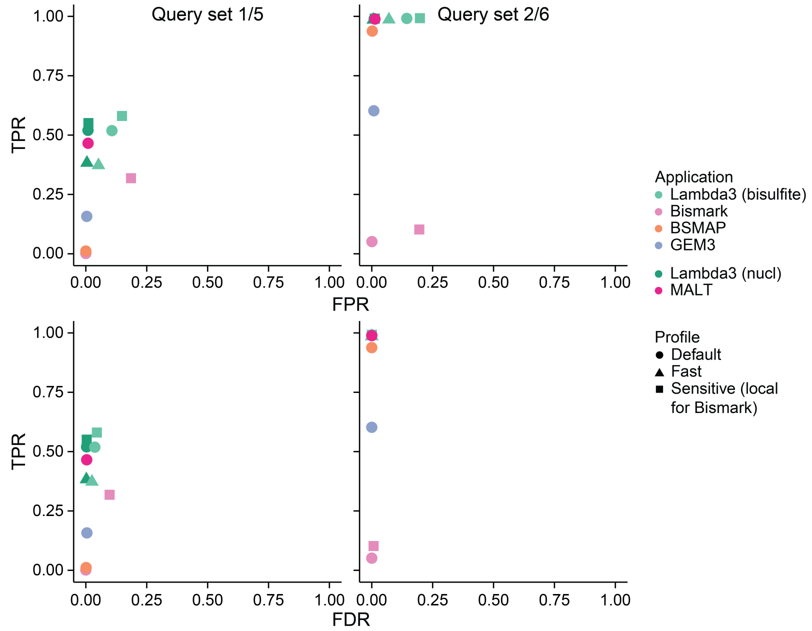

Figure S1: True positive rate (TPR) compared to the false positive rate (FPR, top) and the false discovery rate (FDR, bottom) for bisulfite and nucleotide alignment applications with respect to the results of BlastN with relaxed e-value cut-off.

This showcases that, as expected, local bisulfite alignments are generally more prone to include false positives (Figure S1). Compared to Bismark using local alignments, Lambda3's bisulfite mode detects more true positives ranging from 1.6 to 9.7 fold. The FDR is low for all applications and modes, which stems from the overall small amount of queries not detected by BLAST (Figure S1).

Additional data sets The query data sets q7 and q8 were searched in different databases and no unconverted nucleotide version exists. Therefore, we calculated the bit score threshold based on the matching e-value associated with the bit score of q5 and q6. This led to the bit scores 68 (q5-q7) and 66 (q8) as described in section 3 in the main text.

S2 Performance of the index construction

Index construction is a necessary pre-processing step for almost all applications. It happens once per database file, and does not need to be repeated for subsequent searches. Unless the database is frequently updated, its time and space requirements are not as relevant. We provide the performance measurements here for completeness.

Applications Performance of the index construction for all examined local and semi-global alignment applications was measured using the same infrastructure as for the search. Each application was run with 40 threads except MegaBlast (no parallelisation options available) and Bismark (eight parallel instances were started in line with the search parameters). Only one run was executed for each application.

Results In the protein domain, Diamond is by far the most time- and memory-efficient in creating the index file (Table S1). Lambda3 requires more runtime and memory compared to the previous Lambda2. However, the search performance of Lambda3 improved drastically as described in section 3 in the main text, which outweighs the declines of the index construction as the index only has to be built once for a specific database. Malt requires slightly less memory than Lambda3 but is more than ten times slower than Lambda3 and more than 180 times slower than Diamond. Additionally, Malt builds the largest index, which is almost 500 GiB in size (25 times more space than the database file itself). The index produced by Lambda3 is larger than the index built by Lambda2 but its size can be reduced via compression (Table S1). Again, the index constructed by Diamond is the smallest out of all comparisons (only slightly larger than the actual database).

In the nucleotide domain, MegaBlast produces the smallest index with the least memory and runtime requirements (Table S1). However, it also yields the lowest number of hits per query during the search process, which make the overall application less suitable (see section 3 in the main text). In contrast to the protein domain, Lambda3 needs less than half the runtime of Lambda2 with slightly increased memory consumption and comparable index size. Malt again is slower than any other application and constructs the largest index.

In the bisulfite domain, Lambda3 is the fastest application but requires more memory than the semi-global aligners Bismark and Gem3. Bismark requires the least amount of memory and builds the smallest index while the index constructed by Gem3 is the largest across all comparisons. Bsmap is the only application that does not require an index to be built for the search process.

**Domain**	**Application**	**Runtime (h:mm:ss)**	**Memory (GiB)**	**Size (GiB)**
Protein	Lambda3	35:12	205	59
(20 GiB FastA)	Lambda3 (bgzip)	34:31	215	38
	Lambda2	25:30	126	44
	Diamond	1:50	4	21
	Malt	6:05:16	185	498
Nucleotide	Lambda3	12:27	82	21
(6 GiB FastA)	Lambda2	30:39	61	22
	MegaBlast	46	< 1	2
	Malt	51:38	71	67
Bisulfite	Lambda3	44:02	308	71
(12 GiB FastA)	Bismark	3:10:45	60	64
	Bsmap	-	-	-
	Gem3	2:40:02	205	103

Table S1: Performance of the index construction step for all applications and domains. Bsmap is the only application that does not build an index. Multiple different indexes were built for the bisulfite benchmarks; the numbers shown refer to the largest input file. The value given for "size" is the space occupied by the index file on-disk after construction.

S3 Comparison with Blast

Blast is often considered the "gold standard" of local alignment applications with respect to sensitivity. However, in comparison to other applications, it requires drastically more runtime. For this reason, we could not include Blast in our main benchmarks. To provide at least a superficial comparison, especially with regard to sensitivity, we present a much smaller benchmark in this section. The query sizes were reduced to 2 MiB and 10 MiB for protein and nucleotide search, respectively. It should be noted that the runtime in benchmarks with such small query sizes is not indicative of the runtime on larger datasets (as shown in section 3 in the main text). Especially applications which load large indexes (such as Lambda and Malt) are disproportionately penalised by this setup.

Applications The same infrastructure and parameters of the search were used for all applications that were part of the main benchmarks. Additionally, BlastX and BlastN were included for the protein and nucleotide search, respectively. As Blast does not provide the option to set a maximum bit-score and because e-values are not always comparable across applications (specifically with Blast), we used the following strategy: we executed a single run for each query with an e-value cutoff of 1. We then extracted the maximum e-value associated with the desired minimum bit-score. The actual benchmark was then executed with an e-value threshold corresponding to this measured maximum e-value. As for the other applications, we disabled the composition-based statistics for BlastX in order to ensure comparability of the results.

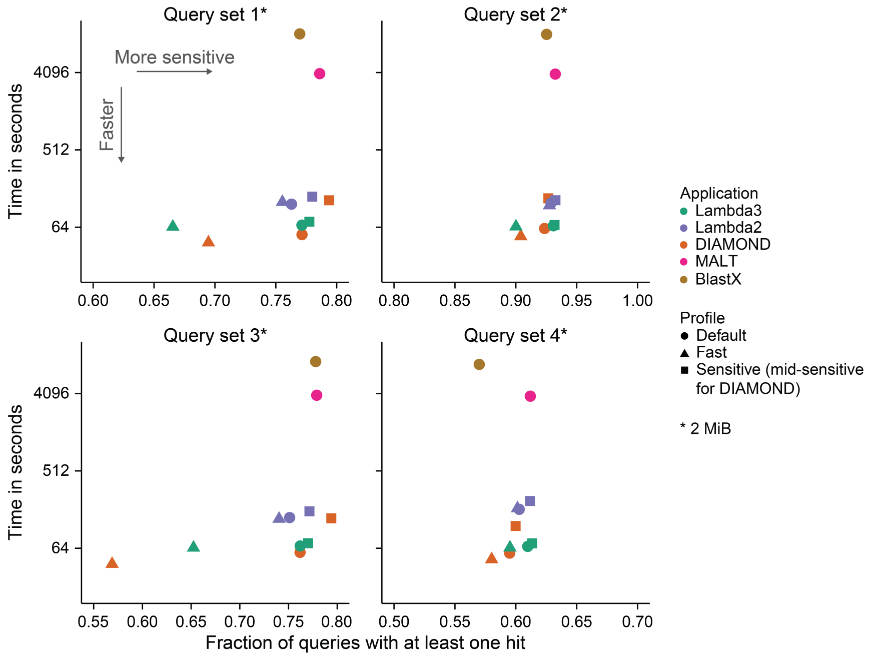

Figure S2: Comparison of local alignment applications for protein search based on runtime and the fraction of detected query sequences of the fastest out of three runs are shown. Subsets of the original query sets were used (2 MiB) in order to ensure the feasibility of a comparison with Blast. Note that the time axis is log-scaled in this figure.

Results In the protein domain, BlastX overall shows similar sensitivity to the default mode of other applications, while the sensitive modes of Diamond and Lambda frequently detect slightly more queries than BlastX (Figure S2). Interestingly, for q4 BlastX detects the least queries compared to any mode of the other applications. When comparing the output of BlastX, Lambda3 and Diamond with more relaxed bit scores/e-values, we found that BlastX finds similar query-alignment pairs, however, with overall shorter length and higher percent identity. These result in lower bit scores (and higher e-values) than the longer alignments detected by the other applications. Overall, as expected, BlastX runs much slower than the other applications (more than 3 hours for Blast searching q1 while Lambda3 and Diamond need around one minute).

In the nucleotide domain, BlastN consistently detects the most queries, clearly outperforming the other applications for q1 and q3 with respect to sensitivity (Figure S3). For q2 and q4, the applications exhibit a more similar sensitivity. Again, BlastN requires the longest runtime (up to 32 times slower than Lambda3's default mode).

S4 Maximising the amount of query-subject pairs

The purpose of Lambda is to find at least one significant hit per query sequence and ideally also "a few" more secondary alignments. This behaviour reflects the most common use-cases where only a limited number of results are helpful. In fact, many users of Blast only search for a single hit (albeit wrongly, Shah et al. [2019]). This choice is implemented through the default setting of ≤ 25 reported hits per query, and is in line with the defaults of programs like Diamond. Other applications may be more appropriate for the task of finding many or even all hits of each query sequence, but we include a comparison here for transparency.

Benchmark setup In this comparison the measure of sensitivity is not *the number of query sequences with at least one hit*, but *the number of unique query-subject pairs*. As explained in section 3.1 in the main text, we do not count more than one hit per query-subject pair, because such hits do not necessarily represent a superior application. The general methodology of the benchmark is the same as for the BlastX benchmarks, however, we chose not to recompute the bit-score threshold based on the size of the small example database, because this would lead to an entirely different sensitivity range from our other benchmarks. Instead we chose to use a minimum bit-score of 47 as in other benchmarks. The results are thus closer to how the applications would perform on larger, real world databases.

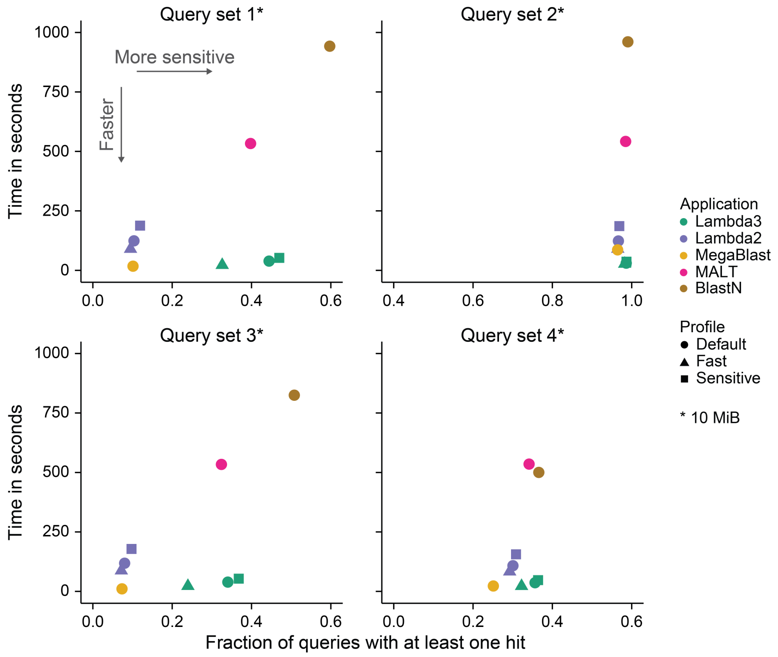

Figure S3: Comparison of local alignment applications for nucleotide search based on runtime and the fraction of detected query sequences of the fastest out of three runs are shown. Subsets of the original query sets were used (10 MiB) in order to ensure the feasibility of a comparison with Blast.

Data sets For known protein sequences, homology search often aims at finding all (or ”very many”) homologues in existing protein databases. This task is akin to clustering and, in contrast to sequence classification, is often performed on smaller untranslated amino acid query data sets. We chose such query and subject datasets from the examples folder[1] of MMseqs2, another local alignment and clustering application (Steinegger and Söding [2017]). The subject file (`DB.fasta`) contains 20000 sequences, and the query file was filtered to only contain the 426 sequences not also present in the former.

Applications Because the scope of this benchmark is very concise, we decided to include, as a reference, an exact aligner that performs full local alignments (Smith-Waterman (SW) algorithm without heuristics) between all input pairs. As a follow-up to the previous section, we included NCBI BlastP, and in addition to the applications compared in the main body of the paper, we also incorporated MMseqs2—which is popular among users of the discussed scenario. Since sensitivity is the primary concern in this comparison, we included more Diamond modes: `sensitive` and `ultra-sensitive`. For Lambda3, we introduce two additional profiles that are more suited to the task of finding many query-subject pairs: `pairs-default` and `pairs-sensitive`. These profiles only perform the second, more sensitive, alignment step.

Results The results are shown in Figure S4. Based on the original goal of finding at least one hit per query sequence, all tested applications perform quite well with different application modes producing between 95% and 100% of the results of the exact algorithm (Figure S4A). BlastP is the most sensitive (100%) with MMseqs2's sensitive mode coming in second. However, MMseqs2 is also the only application to produce a notable number of false-positive hits, i.e. some of the reported alignments have a higher bit-score than those reported by the SW algorithm. This leads to MMseqs2 showing results that other applications correctly discard (effect highlighted in Figure S4). Accounting only for the true positive hits, it still performs very well.

When looking instead at the number of unique query-subject pairs, the sensitivity of the tested applications and settings varies more strongly. Again, BlastP delivers the most results (97%), and MMseqs2's sensitive mode is close behind it (95%, true positives considered). Lambda3 and Diamond deliver much fewer results, especially in their fast and default modes (18% - 39%). The new profiles added for Lambda3 perform much better and similar to Diamond's more sensitive modes (69% - 88%), but they also require notably more time. We conclude that considering both

[1] https://github.com/soedinglab/MMseqs2/tree/master/examples

sensitivity and speed in this scenario—and based on this combination of query and database—, MMseqs2 appears to be a very good choice. Even though Lambda3 was not originally designed for this task, it can easily be configured to achieve the sensitivity of MMseqs2's default mode. We are are optimistic that future versions could incorporate improvements that further increase sensitivity while reducing runtime.

References

S. F. Altschul, W. Gish, W. Miller, E. W. Myers, and D. J. Lipman. Basic local alignment search tool. *J Mol Biol*, 215(3):403–410, Oct 1990.

S. Karlin and S. F. Altschul. Methods for assessing the statistical significance of molecular sequence features by using general scoring schemes. *Proc Natl Acad Sci U S A*, 87(6):2264–2268, Mar 1990.

N. Shah, M. G. Nute, T. Warnow, and M. Pop. Misunderstood parameter of NCBI BLAST impacts the correctness of bioinformatics workflows. *Bioinformatics*, 35(9):1613–1614, May 2019.

M. Steinegger and J. Söding. MMseqs2 enables sensitive protein sequence searching for the analysis of massive data sets. *Nat Biotechnol*, 35(11):1026–1028, Nov 2017.

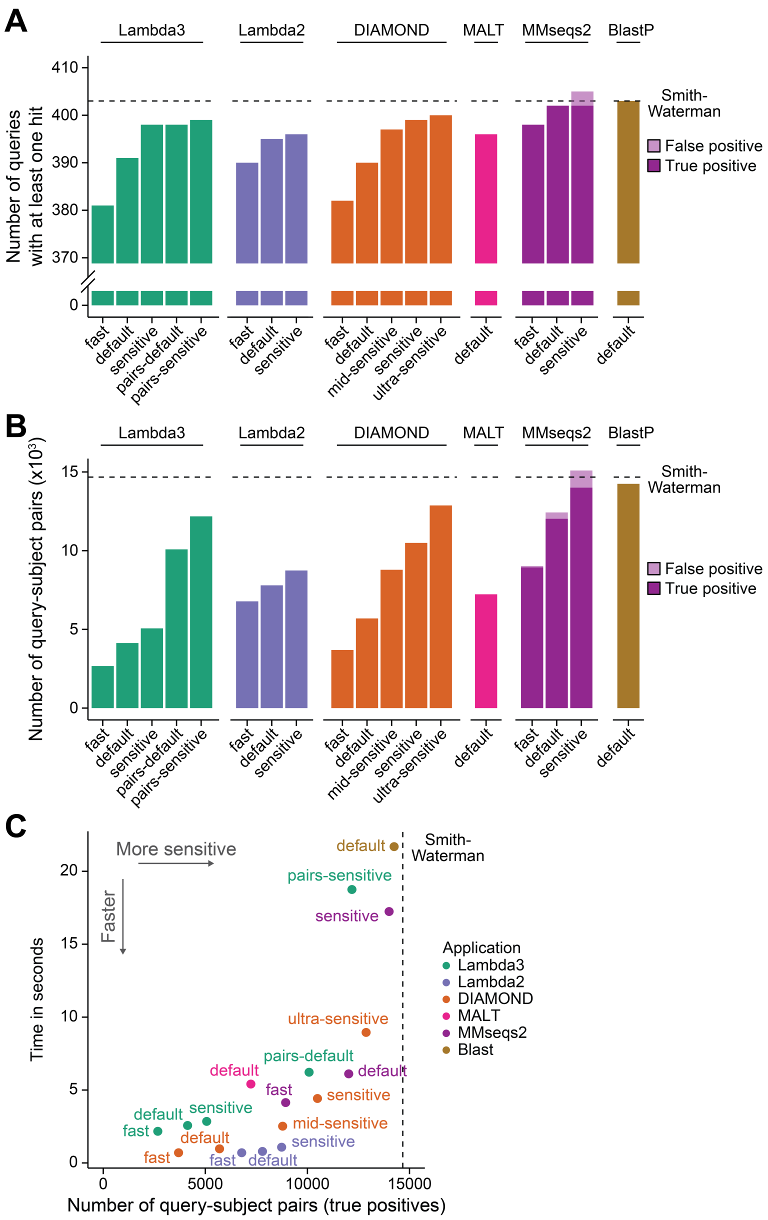

Figure S4: Comparison of local alignment applications for protein search (both query and subject sequences) with the objective to maximise the number of detected query-subject pairs. Due to the small size of the data set, an implementation of the Smith-Waterman algorithm was used as true positive set. A) Number of queries and B) Number of query-subject pairs with at least one hit detected by the different applications. C) Comparison of runtime and the number of true positive query-subject pairs detected by each application.
